# A randomized controlled clinical study on Zuo's acupuncture treatment for prediabetes

**DOI:** 10.1097/MD.0000000000028824

**Published:** 2022-02-25

**Authors:** Xuanli Zeng, Yang Li, Liming Lu, Hao Wen, Guorui Wang, Changbo Zuo

**Affiliations:** a The Second Clinical Medical College, Guangzhou University of Chinese Medicine, China; b Medical College of Acu-Moxi and Rehabilitation, Guangzhou University of Chinese Medicine, China; c Clinical Research and Data Center, South China Research Center for Acupuncture and Moxibustion, Medical College of Acu-Moxi and Rehabilitation, Guangzhou University of Chinese Medicine, China; d Department of Neurology, Sun Yat-sen Memorial Hospital, Sun Yat-sen University, China; e Changbo Zuo International Acupuncture Research Center, Guangzhou University of Chinese Medicine, China.

**Keywords:** prediabetes, prediabetes health education, Zuo acupuncture

## Abstract

**Introduction:**

Prediabetes is a high-risk stage of transition to type 2 diabetes mellitus. Previous studies suggest that acupuncture has potential to prevent prediabetes’ conversion to type 2 diabetes mellitus, which lack of high-quality evidence. Zuo's acupuncture, a kind of acupuncture technique, is formed through long-term and repeated clinical practice by professor Zuo Changbo who integrates the internal meaning of Dong extra acupoints and Taoist medicine principle according to the theories of traditional Chinese medicine. It is used clinically to increase the regression toward normo-glycemi on prediabetes. The objective of this trial is to clarify the clinical effectiveness and safety of Zuo acupuncture for prediabetes.

**Methods and analysis:**

This study is a prospective randomized controlled trial in which 60 patients with prediabetes will be randomly allocated in a 1:1 ratio into either an acupuncture treatment group or a control group. Prediabetes patients in the control group will receive prediabetes health education for lifestyle interventions, whereas patients in the acupuncture group will receive lifestyle interventions plus Zuo Daliji and Yueku stitch treatment. Twenty-four treatment sessions will be performed over 3 months. The primary outcome is conversion rate from prediabetes to normal blood glucose. Secondary outcomes include fasting plasma glucose, 2-hour plasma glucose, glycosylated hemoglobin and blood lipid concentration.

**Ethics and dissemination:**

This study was approved by the Ethics Committee of Guangdong Provincial Hospital of Chinese Medicine (permission number: YF2020-107-01) and the protocol conforms to the principles of the Declaration of Helsinki. Data collection will be completed by June 2022. Publications will be ready for submission in July 2022.

## 1 Introduction

Prediabetes, also known as impaired glucose regulation, is an intermediate metabolic state between normo-glycemia and diabetes, including 2 states of impaired fasting blood glucose and impaired glucose tolerance (IGT), or both.^[1]^ According to a report released by the International Diabetes Federation in 2017, the total number of adults (aged 20–79) diagnosed with type 2 diabetes mellitus (T2DM) is 425 million, accounting for 8.3% of the total population.^[2]^ In addition to overt diabetes, there are 352.1 million people with prediabetes worldwide, which are expected to increase to 531.6 million by 2045.^[3]^ In 2013, the standardized prevalence of diagnosed and undiagnosed diabetes among Chinese adults was 10.9%, while the prevalence of prediabetes was 35.7%.^[4]^ The American Diabetes Association expert panel thought that up to 70% of prediabetic patients will eventually develop diabetes.^[5]^ Relevant data point out that prediabetes is associated with an increased risk of composite cardiovascular events, coronary heart disease, stroke, and all cause mortality.^[6]^ Therefore, controlling the progression of prediabetes which is a reversible condition into diabetes has become a significant public health issue.

Appropriate interventions in the prediabetes stage can prevent and delay the prediabetes’ conversion to T2DM. For prediabetic individuals, lifestyle modification is the cornerstone of diabetes prevention.^[5]^ Measures taken including changes in healthy diet, moderate exercise, regular rest schedule, and relaxed mood. The “Global Diabetes Report”^[7]^ published by the World Health Organization in 2016 pointed out that diet and physical activity interventions are more effective than drugs in preventing diabetes. The earlier the prediabetes get active intervention, the lower the risk of diabetes. Therefore, searching for effective therapies for prediabetes is in demand. However, there are some deficiencies in changing lifestyles, such as insufficient self-discipline, individual differences, limited environmental conditions, and long maintenance time.

As one of the main nondrug therapies, acupuncture has the potential to treat diabetes and prediabetes. Studies have shown that acupoint catgut embedding therapy can improve the conversion rate of prediabetes to normal glucose tolerance, reduce the incidence of diabetes, and have a lasting effect.^[8]^ Although there are few research reports on the treatment of prediabetes with simple acupuncture, there are still many studies on acupuncture to control blood glucose. A pilot randomized placebo-controlled trial have shown that 30 minutes of needling at CV-12 might be useful in reducing blood glucose level in patients with T2DM.^[9]^ A systematic review and meta-analysis of acupuncture for T2DM suggests that acupuncture is beneficial to improving blood glucose control, lipid control and blood pressure control, and weight loss, compared with sham acupuncture or no acupuncture control group.^[10]^

Professor Zuo Changbo comprehended the principles of Dong extra acupoints, combined with Taoism, and established a simple and delicate acupuncture system based on traditional acupuncture. Zuo acupuncture has the advantages of less acupuncture points, simple operation, and no need for acupuncture sensation, which may reduce patients’ pain and fear. Zuo acupuncture has achieved good effect in regulating blood glucose and treating diabetic complications by controlling blood glucose. Zuo Daliji and Yueku stitch are a type of Zuo acupuncture. In a randomized controlled trial, Zhu Jingzhi was given hypoglycemic drugs and methylcobalamin and prostaglandin E1 in the control group (n = 30), and the treatment group (n = 30) combined with Daliji stitch and microcollaterals thorning blood therapy on the basis of oral drugs. The results show that the Daliji stitch combined with micro-collateral puncture can significantly relieve and improve the clinical symptoms of patients with diabetic peripheral neuropathy.^[11]^ Li Yang divided 60 patients with prediabetes into a control group (n = 30) and an acupuncture group (n = 30). The control group was given relevant education to improve lifestyle. The acupuncture group was treated with Zuo Liji stitch based on lifestyle intervention. The result shows that acupuncture Liji stitch can more effectively reverse the prediabetes to normal blood glucose.^[12]^ On the basis of point selection of LiJi stitch, Zusanli (ST36), dacha and Taichong (LR3) are added, which is Zuo Da Li Ji and Yue Ku stitch. This treatment is effective in controlling blood glucose and help restore normal blood glucose levels in patients with prediabetes. However, the exclusive effectiveness of Zuo Daliji and Yueku stitch in individuals with prediabetes is not well documented. The objective of this trial is to clarify the clinical effectiveness and safety of Zuo Daliji and Yueku stitch for prediabetes.

## 2 Methods and analysis

### 2.1 Study design

**Figure 1:**
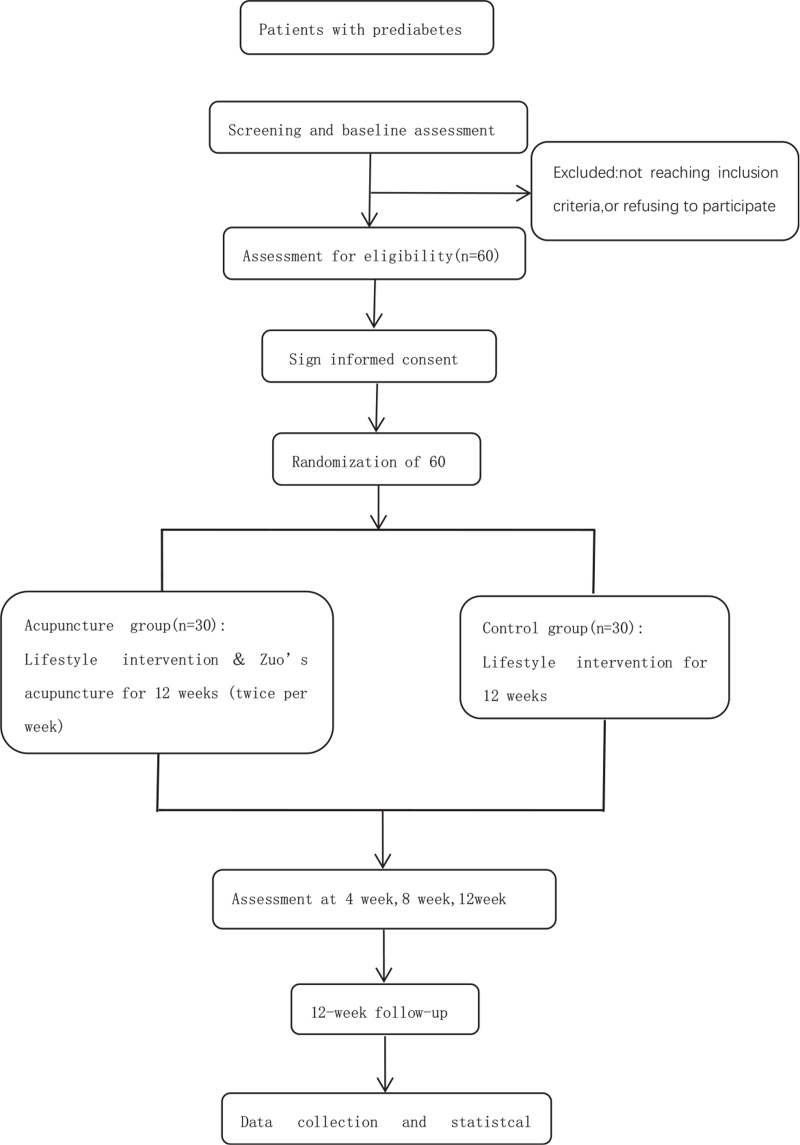
Route diagram of study design.

This study is a prospective randomized controlled trial. It is planned to be conducted from January 1, 2021 to June 1, 2022 in the Traditional Chinese Medicine Hospital of Guangdong Province of China. Prediabetic patients who entered the trial will be randomly assigned to a control group and a treatment group, with 30 cases in each group. Participants in the control group will receive prediabetes health education for lifestyle interventions, whereas patients in the acupuncture group will receive lifestlye interventions plus Zuo Daliji and Yueku stitch treatment. Figure 1 demonstrates the flow of subjects through the trial.

This study protocol conforms to the standard protocol items: recommendations for interventional trials guidelines. Before randomization, all eligible participants will be asked to sign an informed consent.

### 2.2 Sample size

The sample size calculation is based on the 2017 randomized controlled trial report of Ning et al^[8]^ who used acupoint catgut embedding to treat prediabetic patients. In this study, the reversal rate of impaired glucose regulation to Normal Glucose Tolerance is estimated as the outcome. We assume that after 3 months of treatment, the reversal rate of the treatment group is 57% and the reversal rate of the control group is 20%. The test levels (α = 0.05 and β = 0.2) are set. The PASS 2008 software (Beijing HuanZhongRuiChi Technology Co., Ltd.) calculates that the sample size of each group is 25, and 50 in the 2 groups. Assuming 15% drop out, a total of 60 participants will be needed to achieve statistical significance, so each group is required to have 30 participants.

### 2.3 Participants

We will publicize online and post posters to let the public know the details of the research after obtaining ethical approval. Interested patients can contact our research center for more information before participating in the study. Only after the laboratory test meets the selection criteria can they participate. Patients and the public will not be involved with the design, or conduct, or reporting, or dissemination plans of the research.

### 2.4 Inclusion criteria

Participants meeting the following inclusion criteria will be included:

(1) Meet the diagnostic criteria of prediabetes:
  Participants meet one or both of the following criteria can be diagnosed. (According to the WHO criteria for a diagnosis of prediabetes in 1999^[13]^ which is cited by the Guidelines for the prevention and treatment of type 2 diabetes in China [2017 edition]^[10]^):
  **Impaired Fasting Glucose**: 6.1 mmol/L ≤ fasting plasma glucose (FPG) < 7.0 mmol/L, and 2-hour plasma glucose (2-hPG) after a 75 g oral glucose tolerance test (OGTT) <7.80mmol/L.
  **IGT**: FPG < 7.0 mmol/L, and 7.8 mmol/L ≤ 2-hPG (OGTT) < 11.1 mmol/L
(2) Aged 18 to 65 years (either sex)
(3) Voluntary participation and informed consent signed

### 2.5 Exclusion criteria

To reduce the distracting factors of research, participants with any of the following exclusion criteria will be excluded:

(1) Patients who have been taking hypoglycemic drugs and lipid-lowering drugs in the past 3 months
(2) Patients who have received acupuncture treatment in the past 1 month
(3) Patients who have severe fear of acupuncture and refuse acupuncture treatment
(4) Patients with severe cardiovascular and cerebrovascular diseases, liver and kidney dysfunction, blood system diseases or other serious life-threatening diseases, mental illnesses
(5) Pregnant or lactating women
(6) Body mass index ≥ 28 kg/m^2^

### 2.6 Randomization procedure and concealment of allocation

Clinicians will recruit volunteers who meet the research criteria. Patients entering the trial will be randomly assigned to a treatment group or a control group. Randomization will be programmed with SAS 9.2 in the clinical Research and data Center of Guangzhou University of Chinese Medicine. A computer-generated block assigned participants to a treatment or control group on a 1:1 scale. A staff member, independent of this study, will complete the random assignment. The random list will be kept strictly confidential until the study began.

### 2.7 Intervention

A total of 60 patients with prediabetes will be recruited. The patients will be randomly assigned to 2 different groups: the treatment group and the control group. Participants in the control group will receive prediabetes health education for lifestyle interventions for 12 weeks, whereas participants in the acupuncture group will receive lifestyle interventions plus Zuo Daliji and Yueku stitch treatment twice a week for 12 weeks. All of the researchers in the study will receive professional training on prediabetes before participating. All acupuncturists will receive the same training under the clinical treatment plan before the study to standardize the procedures performed by the different acupuncturists. During the trial, in addition to the research interventions, other treatments for blood glucose control will be prohibited.

#### 2.7.1 Lifestyle intervention

Patients in both groups will receive basic health management and lifestyle modification over the course of the 12-week study period. Lifestyle intervention is implemented with reference to the goal of primary prevention of type 2 diabetes in the Guidelines for the Prevention and Treatment of Type 2 Diabetes (2017 Version) in China.^[14]^

The first lecture on prediabetes will be held when the study begins, which is to include the development and prevention of diabetes. Prediabetes health education courses will be performed once a month. Course content includes:

(1) Diet: patients will be instructed to focus on low-sugar, low-salt, light and easily digestible foods, limit the total daily calories intake, reasonably control cholesterol and fat intake, and control body weight.
(2) Living habits: patients will be instructed to work and rest on time. Bad habits such as smoking and drinking will be strictly controlled. Require patients to adhere to 50 minutes of moderate-intensity aerobic exercise at least 3 times per week.
(3) Psychology: patient will be received psychological therapy to relieve patient's pressure, and to inform the patient that the cure or delay of prediabetes progress is likely. It should be treated with a positive and optimistic attitude, and do not be afraid or let it develop.

Researchers will personally understand the patient's lifestyle habits such as diet, rest time and exercise, and give reasonable personalized lifestyle suggestions. The researchers supervised the implementation of the prevention and treatment plan through telephone or Internet follow-up, encouraging family members to participate in intervention management methods to improve the self-management ability of prediabetes patients.

#### 2.7.2 Acupuncture intervention

**Figure 2:**
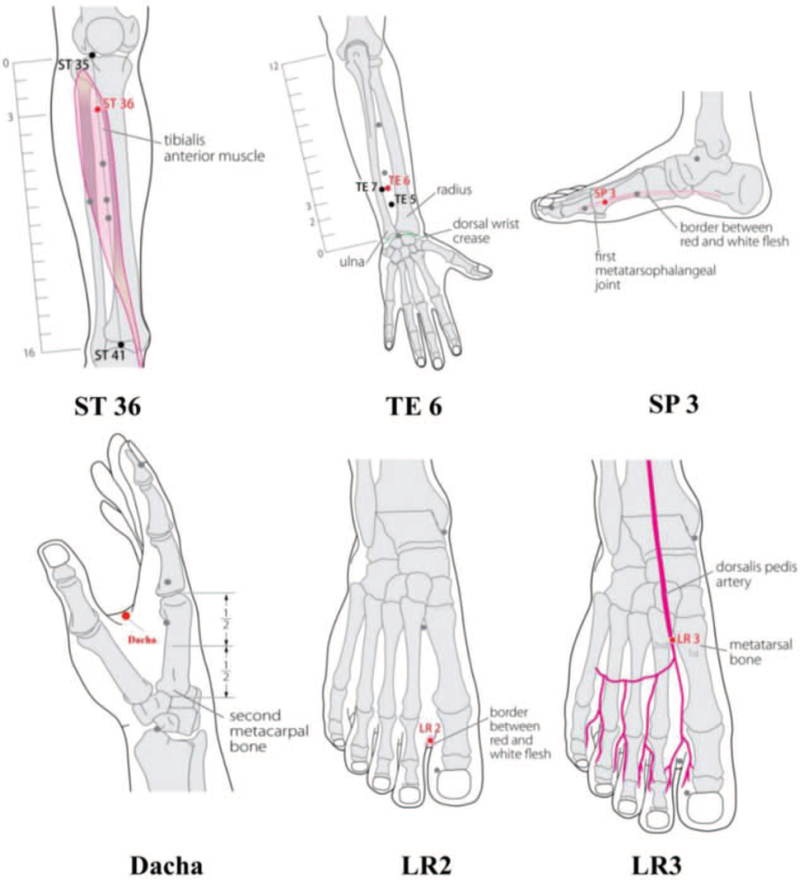
Acupuncture point positioning.

This acupuncture intervention complies with the Standards for Reporting Interventions in Clinical Trials of Acupuncture^[15]^ guidelines. Participants in the treatment group will be treated with Zuo Daliji stitch treatment combined with Yueku stitch treatment. Daliji acupuncture points include Zusanli (ST36), Zhigou (TE6), Taibai (SP3). And Yueku acupuncture points include Taichong (LR3) and Dacha. The location of acupuncture points is shown in Figure 2. The Dacha is a new acupoint discovered by professor Zuo Changbo. It is located between the 1st and 2nd metacarpal bones of the hand and takes the middle point of the red and white meat line with a semi-clenched fist. Disposable, sterile steel, 0.14 × 25 mm or 0.14 × 40 mm acupuncture needles (Eaku disposable acupuncture needles, Maanshan Bond Medical Devices Co, Anhui, China) will be used for the acupuncture treatment. Each needle is equipped with a separate needle insertion tube.

##### 2.7.2.1 Acupuncture manipulation

The patient remained supine, and the researchers routinely disinfected each acupuncture point. The specific operation is as follows.

###### 2.7.2.1.1 Yueku acupuncture treatment

Dacha: Pat the needle gently under the skin at Dacha Point. Remove the needle tube and hold the needle handle and twist the needle gently towards the joint of 1st and 2nd metacarpal bones. At the same time, let the patient breathe slowly and deeply to feel the depth and smoothness of the breath. When the tip of the needle approaches the junction of the first and second metacarpal bones, the needle is raised back into the skin. Operate as described above for 3 to 5 times, and keep the needle.

Taichong (LR3): Gently pat the needle into the skin at a 30° to 45° angle at Xingjian (LR2). Take out the needle tube and hold the needle handle so that the tip of the needle points towards the direction of Taichong Point. After the tip of the needle reaches the Taichong point, the needle is brought back to the skin, and then operated 3 to 5 times as described above, and the needle is left. The patient also needs to breathe slowly and deeply during the acupuncture.

###### 2.7.2.1.2 Daliji acupuncture treatment

The acupuncturists use the above needle insertion method to insert the needle vertically in Zusanli (ST36), Zhigou (TE6), Taibai (SP3), with a depth of about 1 to 2 cm, until the doctor feels that the needle is tightly sucked by the muscle to keep the needle.

**2.7.2.1.2.1 Needle insertion sequence**

The sequence of acupuncture is Taichong (LR3), Dacha, Taibai (SP3), Zhigou (TE6), Zusanli (ST36).

**2.7.2.1.2.2 Needle out sequence**

After taking out the needles in the order of Taichong (LR3), Dacha, Zhigou (TE6), and Zusanli (ST36), let the patient overlap their hands on the lower abdomen, and still breathing slowly and deeply, focusing on Dantian (Qihai (RN6) acupoint). After 5 minutes, take out the needle at Taibai (SP3).

**2.7.2.1.2.3 Treatment course**

The acupuncture session will be for 45 minutes. There will be 2 sessions per week for 12 weeks, giving 24 sessions in total.

### 2.8 Outcome measures

Data collection will be performed by a trained and nonparticipating evaluator who is blinded to the patient's assignment, including at baseline, the intervention phase (4 weeks, 8 weeks, and 12 weeks) and at the end of follow-up (24 weeks).

### 2.9 Primary outcomes

The Primary outcomes is the conversion rate from prediabetes to normal blood glucose. Normal blood glucose means FBG and 2-hPG (OGTT) are in the normal range (FBG < 6.1 mmol/L, 2-hPG < 7.8 mmol/L).

Conversion rate = cases converted to normal blood glucose ÷ total cases of each group ×100%

### 2.10 Secondary outcomes

Secondary outcomes include the following: FPG, 2-hPG after a 75-g OGTT, glycosylated hemoglobin and blood lipid concentration.

FBG and 2h-hPG will be tested at visit 1 (3 days before study), visit 4 (week 12) and the end of follow-up period. Other secondary outcomes will be tested only at visit 1 and visit 4 (Table 1).

**Table 1:**
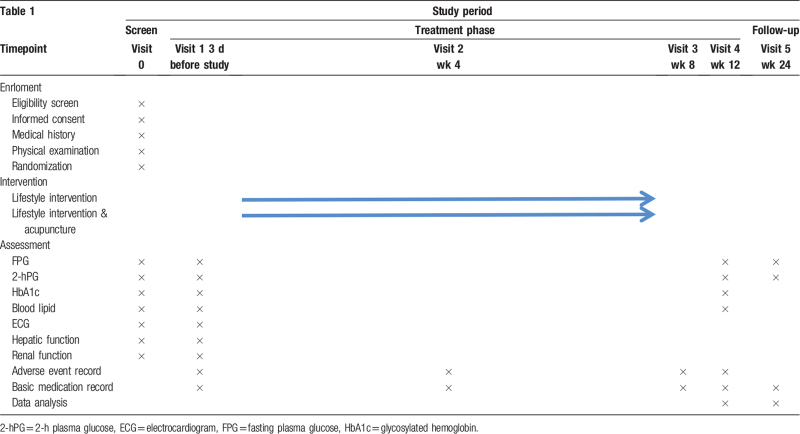
Study timeline according to the standard protocol items: recommendations for interventional trials (SPIRIT) diagram.

## 3 Safety

We will conduct electrocardiogram, liver function, and renal function tests on all participants during the screening phase to exclude patients with severe organic damage. For recording the test period appeared adverse reactions, such as treatment in syncope, local irritation, infection, hemorrhage, stabbing pain unbearable, hysteresis, curved needle, needle acupuncture treatment for sustained severe local pain after 1 hours. Researchers should be on the watch list in cases recorded the degree of symptoms or disease, date of onset, frequency, duration, mitigation, treatment measures and treatment after date, results and follow-up. When an adverse event is found, the observing physician may decide whether to suspend the observation according to the condition. If serious adverse events occur, the unit undertaking the clinical trial shall immediately take necessary treatment measures to protect the safety of the subjects. All adverse events should be followed up and recorded in detail until they are properly resolved or stable.

## 4 Blinding

Owing to the nature of this study, it is not possible to blind the patient or the clinician providing the intervention to the treatment received. The outcome assessor will be blinded to group allocation, unaffiliated with the treatment sites and not involved in providing the interventions.

## 5 Statistical analysis

In this study, the conversion rate from IGT to normal blood glucose between baseline and follow-up period was the main outcome indicator. Statistical analysts will use a one-sided *t* test to compare between the 2 groups. The 2 groups will be compared using the 95% confidence interval method to determine the advantage. Data will be statistically analyzed using PASW Statistics 18.0 (IBM SPSS Inc., Armonk, NY) and SAS 9.2 software (SAS Institute Inc., Cary, NC). For secondary results, a 2-tailed *P* value *<* .5 will be considered statistically significant. For safety evaluation and analysis, first use descriptive analysis, and then use tests to compare the incidence of adverse reactions between the 2 groups. When comparing, consider the severity of adverse reactions and the degree of causation with the intervention. The categorical variables between the 2 groups were compared using Fisher exact test and chi-square test.

## 6 Data management

The case report form (CRF) is filled out by the investigator, and each selected case must complete the case report form. After the completed case report form is reviewed by the inspector, it is handed over to the data administrator for data entry and management. The CRF completed every month will be collected by 2 designated data entry staff within 5 working days for repeated entry, and the final result will be reviewed. When filling out the CRF, researchers should pay attention to the following points: all cases are observed and treated according to the research plan, and the medical record report form is carefully filled out according to the CRF filling requirements. Record the treatment situation and lifestyle intervention of the subjects truthfully. The medical record and the case report form are used as the original records and cannot be changed. The original records cannot be changed when making any corrections. The reason can only be explained by an additional statement, which shall be signed and dated by the doctors participating in the clinical study. After reviewing and confirming the established database, the principal researchers, data managers and statistical analysts will lock the data to ensure the authenticity and reliability of the research results.

## 7 Access to data and confidentiality

Only members of the research team had access to the study data. All research data will be treated in strict confidence. On completion of the study, participants will receive a personal summary of the data collected in the study.

## 8 Trial organisation and monitoring

The investigative team includes the authors listed in this protocol, in addition to 2 other acupuncturists who will assist with subject screening, data collection, subject follow-up and data entry. The principal investigator will manage data flow and perform audits of the procedures, enrolment and treatment throughout the entire process of the study. The associate investigators will monitor the data-collection process and data integrity with periodic evaluation performed continually during the course of the data-collection phase.

## 9 Ethics and patient consent

Ethical approval of this study has been granted by the Ethics Committee of Guangdong Provincial Hospital of Chinese Medicine (permission number YF2020-107-01). And it has been registered in the China Clinical Trial Registration Center (ChiCTR2000035022). Before the start of the study, all subjects will be introduced by the researchers to the purpose and treatment of the study, and signed an informed consent, which includes consent for blood tests. All blood samples taken from participants will be destroyed after the trial. Whether to participate in the study depends entirely on the wishes of the participants, they can withdraw at any time during the study. Participants who have serious adverse reactions or irreversible damage due to the trial will be reasonably compensated as appropriate.

## 10 Discussion

Acupuncture and moxibustion therapies, which make the meridians and collaterals unobstructed and the qi and blood reconciled through stimulating acupoints, so as to achieve the purpose of removing the disease. It has been proven to have a good effect on T2DM patients, and plays an important role in preventing and controlling the occurrence and development of diabetes and its complications.^[16]^ Diabetes has caused severe economic losses and brought a heavy burden to the country and patients.^[17]^ Prediabetes is a critical stage in the development of diabetes. Prediabetes could be treated to prevent progression to diabetes, mitigate some potential consequences of progression to diabetes, and prevent the potential effects of prediabetes itself.

According to the theory of Traditional Chinese medicine, Zuo Changbo believes that the onset of prediabetes is mostly due to the weakening of the promoting effect of Yang qi, which leads the wet phlegm and blood stasis and viscera dysfunction. The acupoint setting of Zuo acupuncture comes from the traditional thoughts of Taoist medicine, which has the function of supporting vital qi, warming viscera and dispelling cold pathogenic factors, so as to regulate the physical condition to treat prediabetes. By comparing the therapeutic effect of combined Zuo acupuncture combined with lifestyle intervention and simple lifestyle intervention on prediabetes, we expect to confirm that acupuncture treatment is more effective in preventing and delaying the development of prediabetes to diabetes, and even reversing normal blood glucose.

## Acknowledgments

We would like to thank all patients and doctors who participated in this trial for their cooperation. We also would like to express our gratitude to my colleagues who contributed their time and effort to the preliminary study (Liming Lu, Yang Li, Xuanli Zeng).

## Author contributions

LY, XLZ, and CBZ conceived and designed the study, collected data, and wrote the manuscript. LML, HW, XLZ, GRW revised the manuscript. LY and XLZ provided ethical advice. CBZ and LML provided methodological suggestions. All authors contributed to the refinement of the research protocol and approved the final manuscript.

**Conceptualization:** Xuanli Zeng, Yang Li.

**Data curation:** Xuanli Zeng, Hao Wen.

**Formal analysis:** Liming Lu.

**Investigation:** Xuanli Zeng, Guorui Wang.

**Methodology:** Liming Lu.

**Writing – original draft:** Xuanli Zeng, Yang Li, Hao Wen, Guorui Wang, Changbo Zuo.

**Writing – review & editing:** Liming Lu, Changbo Zuo.
